# Novel human lymph node-derived matrix supports the adhesion of metastatic oral carcinoma cells

**DOI:** 10.1186/s12885-023-11275-6

**Published:** 2023-08-14

**Authors:** Erika Naakka, Wafa Wahbi, Riia Tiikkaja, Krista Juurikka, Toni Sandvik, Petri Koivunen, Timo Autio, Jukka Tikanto, Janne Väisänen, Hannu Tuominen, Anne Talvensaari-Mattila, Ahmed Al-Samadi, Rabah Soliymani, Pirjo Åström, Maija Risteli, Tuula Salo

**Affiliations:** 1 Department of Oral and Maxillofacial Diseases, Clinicum, Faculty of Medicine, University of Helsinki, Helsinki, Finland; 2 Translational Immunology Research Program (TRIMM), University of Helsinki, Helsinki, Finland; 3 Research Unit of Population Health, Faculty of Medicine, University of Oulu, Oulu, Finland; 4 Medical Research Center, Oulu University Hospital, University of Oulu, Oulu, Finland; 5 Faculty of Biochemistry and Molecular Medicine, University of Oulu, Oulu, Finland; 6 Department of Otorhinolaryngology, Head and Neck Surgery, Oulu University Hospital, Oulu, Finland; 7 Department of Pathology, Oulu University Hospital, Oulu, Finland; 8 Department of Obstetrics and Gynecology, Oulu University Hospital, Oulu, Finland; 9 Institute of Dentistry, School of Medicine, University of Eastern Finland, Kuopio Campus, Kuopio, Finland; 10 Meilahti Clinical Proteomics Core Facility, Faculty of Medicine, HiLIFE, University of Helsinki, Helsinki, Finland; 11 Research Unit of Biomedicine, Faculty of Medicine, University of Oulu, Oulu, Finland; 12 Biocenter Oulu, Oulu, Finland; 13 Department of Pathology, HUSLAB, Helsinki University Central Hospital, University of Helsinki, Helsinki, Finland

**Keywords:** Myogel, Lymphogel, Lymph nodes, Oral tongue squamous cell carcinoma, Tumour microenvironment

## Abstract

**Background:**

3D culture is increasingly used in cancer research, as it allows the growth of cells in an environment that mimics in vivo conditions. Metastases are the primary cause of morbidity and mortality in cancer patients, and solid tumour metastases are mostly located in lymph nodes. Currently, there are no techniques that model the pre-metastatic lymph node microenvironment in vitro. In this study, we prepared a novel extracellular matrix, Lymphogel, which is derived from lymph nodes, mimicking the tumour microenvironment (TME) of metastatic carcinoma cells. We tested the suitability of the new matrix in various functional experiments and compared the results with those obtained using existing matrices.

**Methods:**

We used both commercial and patient-derived primary and metastatic oral tongue squamous cell carcinoma (OTSCC) cell lines. We characterized the functional differences of these cells using three different matrices (human uterine leiomyoma-derived Myogel, human pre-metastatic neck lymph node-derived Lymphogel (h-LG), porcine normal neck lymph node-derived Lymphogel (p-LG) in proliferation, adhesion, migration and invasion assays. We also performed proteomic analyses to compare the different matrices in relation to their functional properties.

**Results:**

OTSCC cells exhibited different adhesion and invasion patterns depending on the matrix. Metastatic cell lines showed improved ability to adhere to h-LG, but the effects of the matrices on cell invasion fluctuated non-significantly between the cell lines. Proteomic analyses showed that the protein composition between matrices was highly variable; Myogel contained 618, p-LG 1823 and h-LG 1520 different proteins. The comparison of all three matrices revealed only 120 common proteins. Analysis of cellular pathways and processes associated with proteomes of each matrix revealed similarities of Myogel with h-LG but less with p-LG. Similarly, p-LG contained the least adhesion-related proteins compared with Myogel and h-LG. The highest number of unique adhesion-related proteins was present in h-LG.

**Conclusions:**

We demonstrated that human pre-metastatic neck lymph node-derived matrix is suitable for studying metastatic OTSCC cells. As a whole-protein extract, h-LG provides new opportunities for in vitro carcinoma cell culture experiments.

**Supplementary Information:**

The online version contains supplementary material available at 10.1186/s12885-023-11275-6.

## Background

Although 2D culture settings on plastic are simple and convenient to use, they have many critical limitations when aiming to replicate the complexity and pathophysiology of a tumour. Under such simplified conditions, tissue-specific architecture, mechanical and biochemical signals and cell-cell and cell-matrix interactions are lost [1]. Cell adhesion molecules play a role in cell-cell and cell-extracellular matrix (ECM) interactions [2], but since ECM adhesions by cells to different components of the tumour microenvironment (TME) cannot be established on plastic surfaces, classical cell culture conditions do not adequately represent the in vivo situation. Three-dimensional (3D) cell culture models provide a higher level of physiological resemblance to the human tumour microenvironment (TME) compared to conventional two-dimensional (2D) cell culture models. In 3D, the cells’ gene expression is more comparative to the in vivo and the cell shape and polarization is preserved [3]. 3D cell culture models also offer improved accuracy in evaluating the effectiveness and appropriate dosage of anti-cancer medications [4, 5] and possible resistance to therapeutics [6].

Cancer research, including drug testing, has traditionally focused mostly on cancer cells originating from primary tumours. However, in many solid cancers, metastases are the main cause of cancer deaths, and the availability of efficient drugs to prevent or treat metastatic tumours is very limited [7]. Despite the availability of multiple ECM mimics from various sources [8], a lymph node (LN)-derived matrix has remained unavailable. New 3D culture materials mimicking in vivo tissue composition are needed, for example, to bridge the gap between preclinical and clinical drug testing.

We formerly developed the first human tumour-derived matrix, Myogel [9]. It is an ECM protein extract generated from human uterine leiomyoma tumour tissue as a by-product of surgical operations. Myogel is an excellent tool for testing human carcinoma cells in vitro, and it has been successfully used for culturing several cell lines in numerous studies [10–17]. As an example, Myogel, as compared with plastic and mouse sarcoma–derived Matrigel, promotes the growth of freshly isolated cancer cells from primary tumours [12] and improves the predictability of head and neck cancer drug testing [18].

Of head and neck cancers, oral tongue squamous cell carcinoma (OTSCC) is the most prevalent cancer in the oral cavity, and despite improvements in treatment, it remains characterized by poor survival, mostly because of its tendency for LN metastasis [19]. However, it is not well understood how metastatic cells and their TME differ from tumour cells and the TME of the primary tumour site. In this study, we characterized the functional differences of primary and metastatic OTSCC cells in different TME mimicking matrices to determine whether metastatic cells prefer the LN-based environment. In addition to the previously reported human leiomyoma-derived matrix Myogel [9], we produced and tested the novel ECM matrices of porcine Lymphogel (p-LG), prepared from normal porcine LNs, and human Lymphogel (h-LG), prepared from head and neck squamous cell carcinoma (HNSCC) patients’ pre-metastatic LNs, and compared the protein composition of these matrices. We showed that Myogel and h-LG could be used for OTSCC cell studies to mimic the TME of primary and metastatic tumour cells, respectively.

## Methods

### Cell lines and cell culture

The experimental protocol for use of OTSCC patient samples was approved by the Ethics Committee of the Northern Ostrobothnia Hospital District, Finland (statement no. 31/2016). Four primary (OU-OTSCC-7A, OU-OTSCC-9A, OU-OTSCC-18A, OU-OTSCC-15A) and three metastatic (OU-OTSCC-7B, OU-OTSCC-9B, OU-OTSCC-18B) tumour cell lines were isolated from OTSCC patient samples at Oulu University Hospital after informed consent. Clinical characteristics of OU-OTSCC cell lines are described in Additional file 1: Table S1. The fresh tumour pieces were washed thoroughly in PBS containing 100 U/ml penicillin, 100 µg/ml streptomycin and 2.5 mg/ml amphotericin B (all from Merck, Darmstadt, Germany) and minced with a razor blade into small (1–2 mm) pieces. After washing with PBS, tissues were digested in 1:1 Dulbecco´s Modified Eagle Medium (DMEM)/Ham´s Nutrient Mixture F 12 (Thermo Fisher Scientific, Waltham, MA, USA) supplemented with 100 units/ml penicillin, 100 µg/ml streptomycin, 250 ng/ml amphotericin B and 1 mg/ml collagenase (all from Merck) for about 1 h at 37 °C with stirring. Single cells were obtained by filtrating the digestion with a 40 μm cell strainer (Corning inc., Corning, NY, USA). Single cells were seeded on 12-well culture plates in minimal essential medium (MEM, Thermo Fisher Scientific) supplemented with 10% heat-inactivated FBS (Thermo Fisher Scientific), 1% non-essential amino acids (Thermo Fisher Scientific), 2 mM glutamine, 100 U/ml penicillin, 100 µg/ml streptomycin and 250 ng/ml amphotericin B (all from Merck). Fibroblasts were removed by brief exposure to trypsin-EDTA solution (Merck), as previously described [16].

UT-SCC-24A (primary tongue cancer, RRID:CVCL_7826), UT-SCC-24B (metastatic tongue cancer, RRID:CVCL_7827), UT-SCC-42A (primary larynx cancer, RRID:CVCL_7847) and UT-SCC-42B (metastatic larynx cancer, RRID:CVCL_7848) have been established at the Department of Otorhinolaryngology – Head and Neck Surgery, Turku University Hospital, Finland and were kindly provided by Professor Reidar Grénman. UT-SCC and the commercial human OTSCC HSC-3 (Japanese Collection of Research Bioresources (JCRB) Cell Bank, JCRB0623) cell lines were cultured in DMEM/F-12 supplemented with 10% heat-inactivated FBS, 100 U/ml penicillin, 100 µg/ml streptomycin (all from Thermo Fisher Scientific), 0.4 µg/ml hydrocortisone (Merck), 50 µg/ml ascorbic acid (AppliChem GmbH, Darmstadt, Germany) and 250 ng/ml amphotericin B (Merck). All cell lines were maintained at 37 °C with 5% CO_2_ and 95% humidity. All the cell lines used were at passage number under 30.

### Preparation of Myogel and Lymphogel

Experimental protocols on human leiomyoma tissue were approved by the Ethics Committee of the Northern Ostrobothnia Hospital District, Finland (statement no. 2/2017). Clinically, non-metastatic human cervical lymph node tissues (herein, pre-metastatic LNs) were collected from three oral or hypopharyngeal cancer patients undergoing neck dissection surgery. Based on the clinical evaluation, the patients had more than two metastases. The collected fresh tissue was evaluated to be macroscopically non-metastatic by the expert pathologist. The study was approved by the Ethics Committee of the Northern Ostrobothnia Hospital District, Finland (statement no. 31/2016). All patient samples were collected after informed consent was obtained.

Normal anterior cervical lymph nodes were dissected from healthy domestic pigs (*Sus scrofa domesticus*) sacrificed for other experimental study at the University of Oulu Laboratory Animal Centre KEKS (study protocol approved by internal licenses 14/2017 and 26/2019). The animals were purchased for the experimental study from a contract breeder (Aprakka Oy, Utajärvi, Finland) providing pigs for Oulu Laboratory Animal Centre. The original experimental study protocol was reviewed and approved by the National Animal Experiment Board of Finland (ESAVI/6941/04.10.07/2016) and animals were sedated with midazolam 0.2 mg/kg (Midazolam Hameln; Hameln Pharma GmBH, Hameln, Germany) and ketamine 10 mg/kg (Ketaminol vet; Intervet International B.V., Boxmeer, The Netherlands) combination. Anesthesia was induced with fentanyl (50 µg) (Fentanyl Hameln; Hameln Pharma GmBH) and maintained with sevoflurane (1-1.5%) (Sevorane; AbbVie S.r.l, Roma, Italy) and infusion of fentanyl and midazolam. After surgical operations and experiment, the animals were euthanized with pentobarbital-Na 60 mg/kg (Mebunat vet; Orion Oyj, Espoo, Finland).

Myogel was prepared according to Salo et al. [9]. For preparation of Lymphogel, minor modifications were generated to the Myogel protocol. Briefly, tissue powder was suspended in NaCl buffer (3.4 M NaCl, 25 mM Tris-HCl pH 7.4, 4 mM EDTA, 2 mM N-ethylmaleimide, all from Merck), centrifuged, re-suspended and homogenized in urea buffer (2 M urea, 0.05 M Tris-HCl pH 7.4, 0.154 M NaCl, all from Merck). Further processing of Lymphogel was performed according to Salo et al. [9]. DC Protein Assay (Bio-Rad) was used to measure the protein concentration.

### Myoma invasion assay

Myoma organotypic cultures were prepared as previously described [20, 21]. Briefly, 7 × 10^5^ OU-OTSCC and HSC-3 cells in 50 µl of medium were added to the top of the myoma disc in Transwell® inserts (diameter 6.5 mm; Corning). The next day, the discs were transferred onto steel grids in 12-well plates with 1 ml of medium. Cells were cultured on top of the myoma disc for 18–20 days. The tissues were fixed in 4% neutral-buffered formalin, dehydrated and embedded in paraffin. Then 6 μm sections were prepared for immunohistochemistry with monoclonal pan-cytokeratin AE1/AE3 antibody (1:150, Dako, Glostrup, Denmark) as described previously [22]. Images were acquired from the sections with a DM4000B microscope connected to a DFC-320 camera using QWin V3 software (all from Leica Microsystems, Wetzlar, Germany).

### Viability assay

96-well plates (Corning) were coated with 1 mg/ml of each matrix: Myogel, porcine Lymphogel (p-LG) or human Lymphogel (h-LG) and incubated at 37 °C overnight. Next 7000 cells in 100 µl of complete medium was dispensed into each well and incubated for 3 days at 37 °C. Resazurin sodium salt (Merck) was added at a final concentration of 3 µg/ml, and fluorescence was analysed after 3 h using 544/15 (excitation) and 595/60 nm (emission) filters in the Victor3V 1420 Multilabel Counter equipment (Perkin Elmer Life, Waltham, MA, USA). Results represent the average of three independent experiments, performed in quintuplicates.

### Adhesion assay

96-well plates (Corning) were coated as described above. The following day, the wells were washed with PBS, and 6000 cells were seeded into 100 µl of serum-free medium. After a 2 h incubation at 37 °C, the non-adherent cells were rinsed off with PBS, and the remaining cells were fixed with 10% (w/v) trichloroacetic acid, washed with H_2_O, air-dried overnight and stained with 0.1% (w/v) crystal violet solution (Merck). Finally, the cells were imaged with a PowerShot S50 (Canon, Tokyo, Japan) camera connected to an Eclipse TS100 (Nikon, Tokyo, Japan) microscope and counted using Fiji software [23]. Results represent the average of three independent experiments, performed in quintuplicates.

### Spheroid invasion assay

Spheroid invasion assay was conducted according to Naakka et al. [7]. For spheroid generation, 1000 cells were seeded into ultra-low attachment 96-well plate (Corning) wells in 50 µl of complete medium and incubated for 4 days at 37 °C. Next, the spheroids were embedded in 50 µl of matrix in complete culture medium of each cell line. The resulting final matrix concentrations were as follows: 0.5 mg/ml Myogel, p-LG or h-LG, 0.5 mg/ml Fibrinogen (Merck), 0.3 U/ml Thrombin (Merck) and 33.3 µg/ml Aprotinin (Merck). The plate was then incubated at 37 °C for 30 min to allow the matrix to solidify and 100 µl of complete culture medium was added on top of the matrix. Spheroids were imaged at 0 h and after 4 days of incubation at 37 °C, using a Nikon Eclipse TS100 (Nikon) inverted light microscope, with 4x magnification, connected to a Canon PowerShot S50 (Canon) camera. Fiji software was used for measuring the area covered by spheroids [23]. The fold-change in total spheroid area at day 4 compared with the area at 0 h was calculated. Results represent the average of three independent experiments, performed in sextuplicates.

### Scratch wound cell migration and invasion assays

IncuCyte 96-well ImageLock Microplate wells (Sartorius, Göttingen, Germany) were coated with 300 µg/ml Myogel, p-LG or h-LG for migration and invasion assays and incubated at 37 °C in a cell culture incubator overnight. The excess of the gels was removed before seeding the cells. For both assays, the cells were seeded at a density of 25,000 cells per well in 100 µl of complete medium. After 24 h at 37 °C, a 96-pin IncuCyte WoundMaker Tool (Sartorius) was used to make uniform wounds on the confluent monolayer of the cells. The wells were washed two times with medium, and 100 µl of complete medium was added. For the invasion assay, 50 µl of Myogel-collagen or Lymphogel-collagen matrix (2.4 mg/ml Myogel/p-LG/h-LG and 0.8 mg/ml type I rat tail collagen) (Corning) was added on top of the cells. After the matrix was solidified, 50 µl of complete media was added, and the plates were transferred to an incubator. The wound closing was monitored automatically every 3 h for 24 h using IncuCyte S3 Live-Cell Imaging System (Sartorius). Analysis of wound closing (width of the wound) was performed using Ilastik (freeware) [24] and Fiji software [23]. Results represent the average of three independent experiments, performed in quadruplicates.

### Proteolytic digestion of matrices and proteomic analysis

The proteomic analyses of Myogel and both Lymphogels were performed according to Scifo et al. [25]. A total of 10 µg of proteins were reduced by 10 mM DTT in 100 mM ammonium bicarbonate (30 min at room temperature) and alkylated with 50 mM iodoacetamide in 100 mM ammonium bicarbonate (15 min at room temperature in the dark). Trypsin solution was added in a ratio of 1:50 w/w to 50 mM ammonium bicarbonate. The peptide samples were cleaned using C18-reverse phase ZipTip (Merck) and dried after elution. Before injection into LCMS, the peptide digests were resuspended in 1% TFA and sonicated in a water bath for 1 min.

Of digested proteins, 300 ng were injected for LC-MS^E^ analysis. The peptides were separated using nanoAcquity UPLC system (Waters Corp., Milford, MA, USA) equipped with a Symmetry C18 reversed-phase trapping column (180 μm × 20 mm, 5 μm particles; Waters) and an analytical BEH-130 C18 reversed-phase column (75 μm × 250 mm, 1.7 μm particles; Waters) in a single pump trapping mode. The injected sample analytes were trapped at a flow rate of 15 µl/min in 99.5% of solution A (0.1% formic acid) and then separated with a linear gradient of 3–35% of solution B (0.1% formic acid/acetonitrile), for 100 min at a flow rate of 0.3 µl/min and a stable column temperature of 35 °C.

The samples were run in ion mobility assisted in data-independent analysis mode (HDMSE), in a Synapt G2-S mass spectrometer (Waters), by alternating between low collision energy (6 V) and high collision energy ramp in the transfer compartment (20 to 45 V) and using a 1-sec cycle time. The separated peptides were detected online with a mass spectrometer, operated in positive resolution mode in the range of *m/z* 50-2000 amu. For lock mass correction, human [Glu^1^]-fibrinopeptide B (150 fmol/µl, Merck) in 50% acetonitrile/0.1% formic acid solution at a flow rate of 0.3 µl/min was applied every 30 s.

Protein identification relative quantification between samples using precursor ion intensities was performed with Progenesis QI for Proteomics™ Informatics for Proteomics software (Nonlinear Dynamics, Waters). MSE parameters were set as follows: low energy threshold of 135 counts, elevated energy threshold of 30 counts, and intensity threshold of precursor/fragment ion cluster 750 counts.

Database searches were carried out against Human Proteome UniProtKB-SwissProt database (release 2019_11, 74,807 entries) with Ion Accounting algorithm and using the following parameters: peptide and fragment tolerance: automatic. Maximum protein mass: 750 kDa. Minimum fragment ions matches per protein ≥ 7. Minimum fragment ions matches per peptide ≥ 3. Minimum peptide matches per protein ≥ 1. Primary digest reagent: trypsin. Missed cleavages allowed: 2. Fixed modification: carbamidomethylation C. Variable modifications: deamidation of NQ residues. Oxidation of Methionine and false discovery rate (FDR) < 4%.

### Statistical and bioinformatic analyses

Statistical analyses were carried out with SPSS Statistics 27 (IBM Corp., Armonk, NY, USA). To determine the statistical significance, we performed with one-way analysis of variance (ANOVA) followed by Bonferroni correction or independent samples T-test. P-value < 0.05 was considered statistically significant. Figures were created with Origin 2020 graphing software (OriginLab Corp., Northampton, MA, USA) and Adobe Photoshop CC 2019 (Adobe Inc., San Jose, CA, USA).

Three technical replicates for each matrix were produced by proteomic analysis. To study the proteomes of each matrix, the data from replicates were combined, and duplicates were removed based on the protein accession number. Next, uncharacterized proteins along with proteins with duplicated descriptions (Names) were removed for each matrix. Proteins found in each matrix were analysed with Metascape [26] to identify the biological processes enriched in each matrix. Analysis settings were: Minimum overlap was set to 3, minimum enrichment was set to 3 and p-value cutoff at 0.01. MetaScape analysis uses various databases such as STRING, KEGG and Reactome (full list available at https://metascape.org/) and can be used to assess e.g., enrichment of biological and cellular pathways, drug targets and diseases of a user-provided list of genes/proteins. The total proteomes of each matrix were compared to identify common and unique proteins.

The proteins (by protein entry) and intensities (by matched product) identified by proteomic analysis for three technical replicates of Myogel and h-LG were combined. For quality control and to compare levels of proteins present in both matrices, the proteins present in only one technical replicate or in only one matrix were filtered out. Missing single values were replaced by the average intensity of the two other technical replicates. The intensity values for each protein entry were log2-transformed and normalized with quantile normalization method. The mean transformed and normalized intensity values for each protein entry were compared between matrices, and a fold-change of 2 was used as the threshold to identify differentially expressed proteins between matrices. R version 4.2.2 with “dplyr”,”data.table” and ”zoo” packages were utilized for this analysis.

Cell adhesion-related proteins common and unique to each matrix were extracted from the total proteome of each matrix by a comparison with proteins associated with the GO-term GO:0007155 (Cell Adhesion) in the GeneOntology (GO) database. Shared proteins and adhesion proteins with similar names and different accession numbers between human and porcine matrices were compared based on sequence identity on the BLAST tool from NCBI (Bethesda, MD, USA). Information on the cellular location for adhesion-related proteins were collected from UniProt via UniProt ID mapping tool [27].

## Results

### Effect of matrix on cell viability varies without any clear difference

**Fig. 1:**
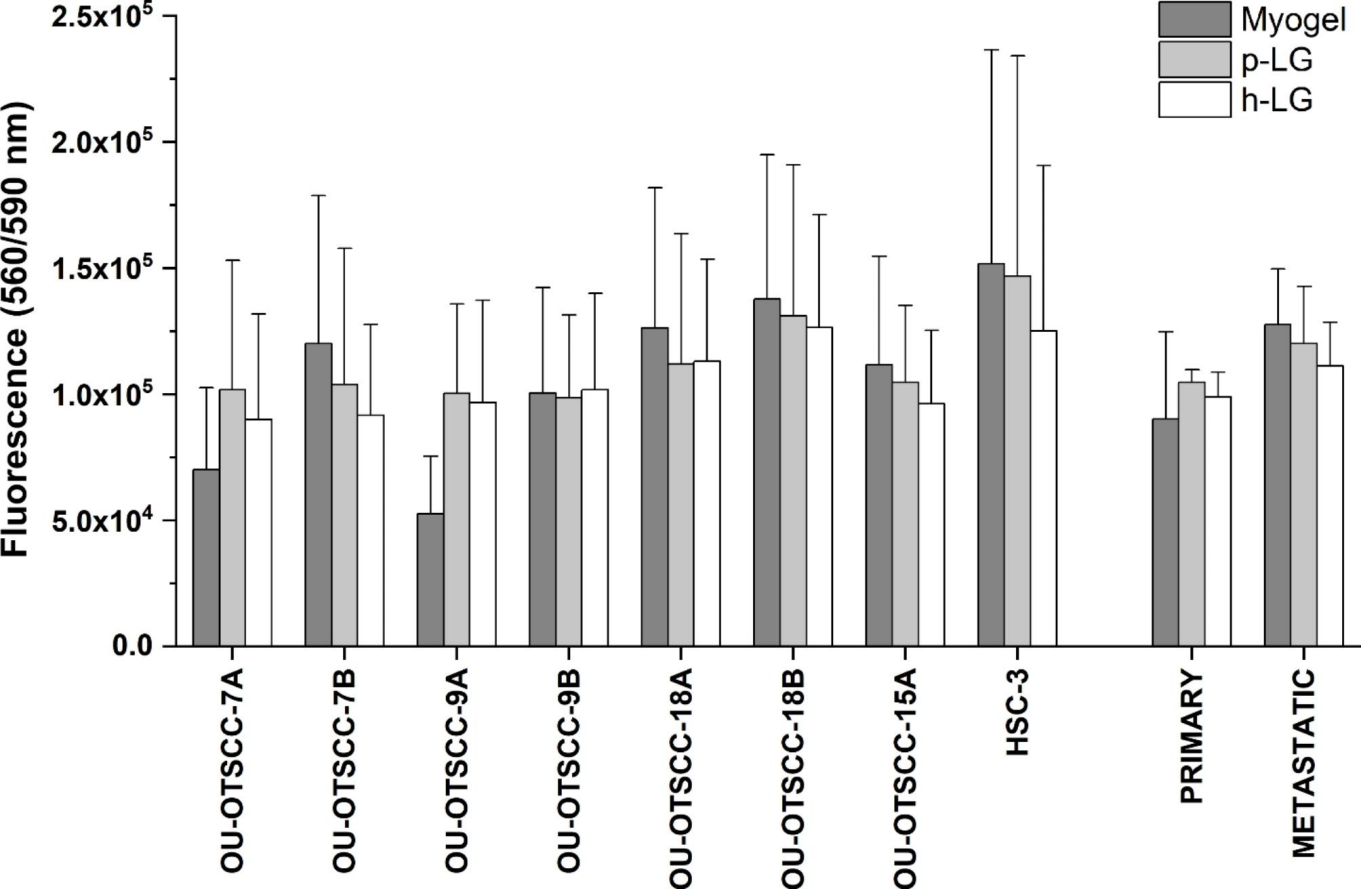
Viability of OU-OTSCC and HSC-3 cell lines on different matrices. Cells were cultured 72 h in wells coated with Myogel, porcine Lymphogel (p-LG) and human Lymphogel (h-LG). Cell viability was analysed with resazurin assay. The values of the cell lines represent the average ± SD of three independent experiments. For “primary” and “metastatic” bars, the average results ± SD of four cell lines were combined. P-values were calculated by one-way analysis of variance (ANOVA) followed by Bonferroni correction or independent samples T-test

OU-OTSCC cell lines established from primary and metastatic tumours showed pancytokeratin immunostaining similar to HSC-3 cells, confirming that all cell lines are epithelial in origin and considered as carcinoma cell lines (Additional file 2: Fig. S1). Cell viability was measured to examine whether the matrices have different effects on the viability of the primary and metastatic cell lines and could thereby affect other cell functions examined. All cell lines were viable in all tested matrices. The viability mostly varied between the cell lines and did not depend on the matrices (Fig. 1).

### Metastatic cells adhere more to h-LG, primary cells more to Myogel

**Fig. 2:**
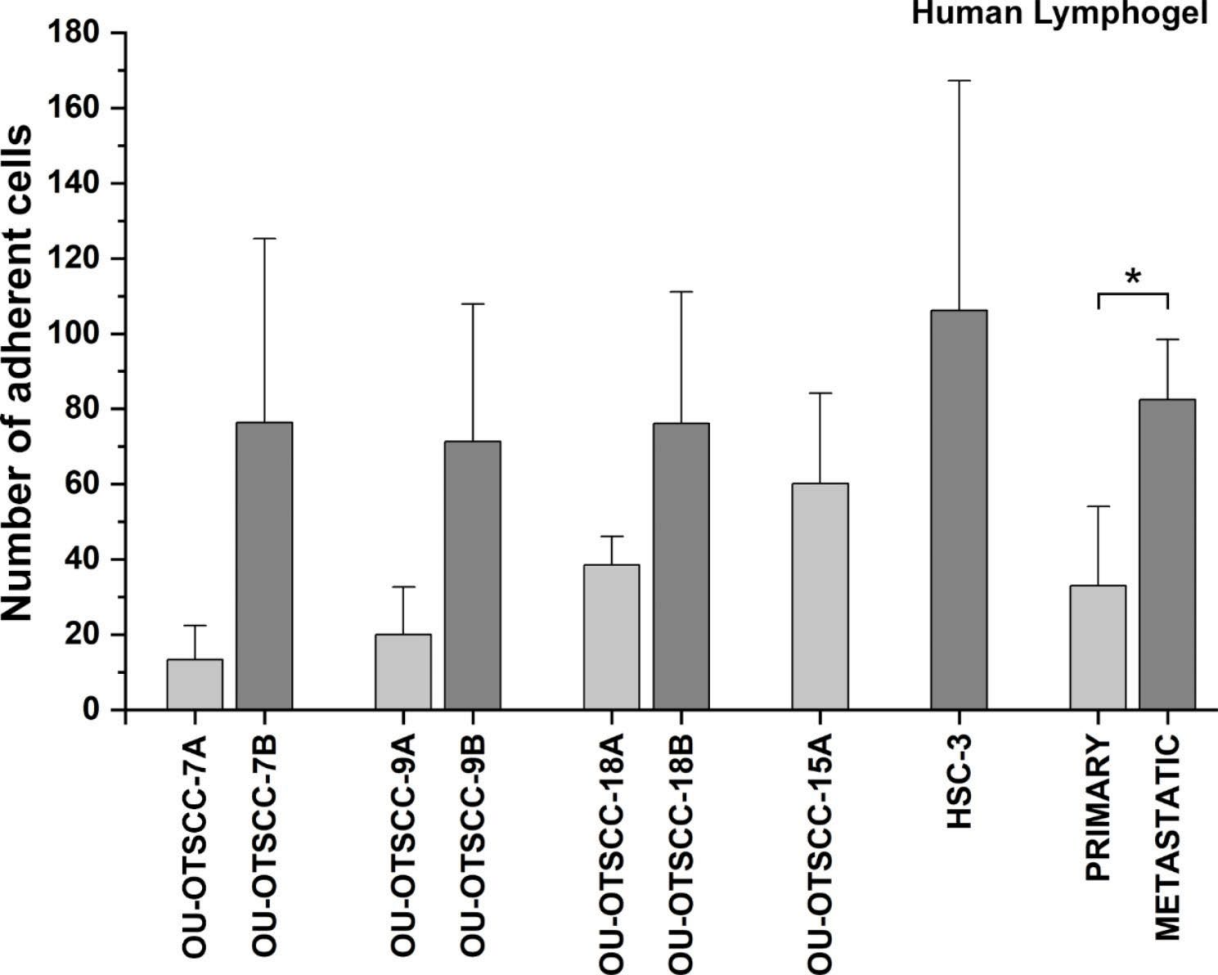
Adhesion of OU-OTSCC and HSC-3 cell lines on human Lymphogel. Cells were allowed to attach for 2 h in wells coated with human Lymphogel, after which cells were fixed, stained with crystal violet and counted. The values of the cell lines represent the average ± SD of three independent experiments. For “primary” and “metastatic” bars, the average results ± SD of four cell lines were combined. P-values were calculated with Independent Samples T-test. ^*^P < 0.05

A cell-substrate adhesion assay was performed to examine whether primary and metastatic cell lines performed differently on the tested matrices. Approximately the same number of cells from all cell lines adhered to Myogel coating. To the wells coated with p-LG, a variable number of cells adhered without any clear pattern in adhesion (Additional file 2: Fig. S2). Interestingly, all tested metastatic cell lines adhered significantly more to the wells pre-coated with h-LG when compared with adhesion with the corresponding primary cell lines derived from the same patients (Fig. 2). Metastatic HSC-3 cell line also adhered more than primary OU-OTSCC-15 A cell line to h-LG. All primary cell lines adhered more to Myogel than to either of the Lymphogel matrices (Additional file 2: Fig. S2).

### For invasion, primary and metastatic cells prefer different matrix types

**Fig. 3:**
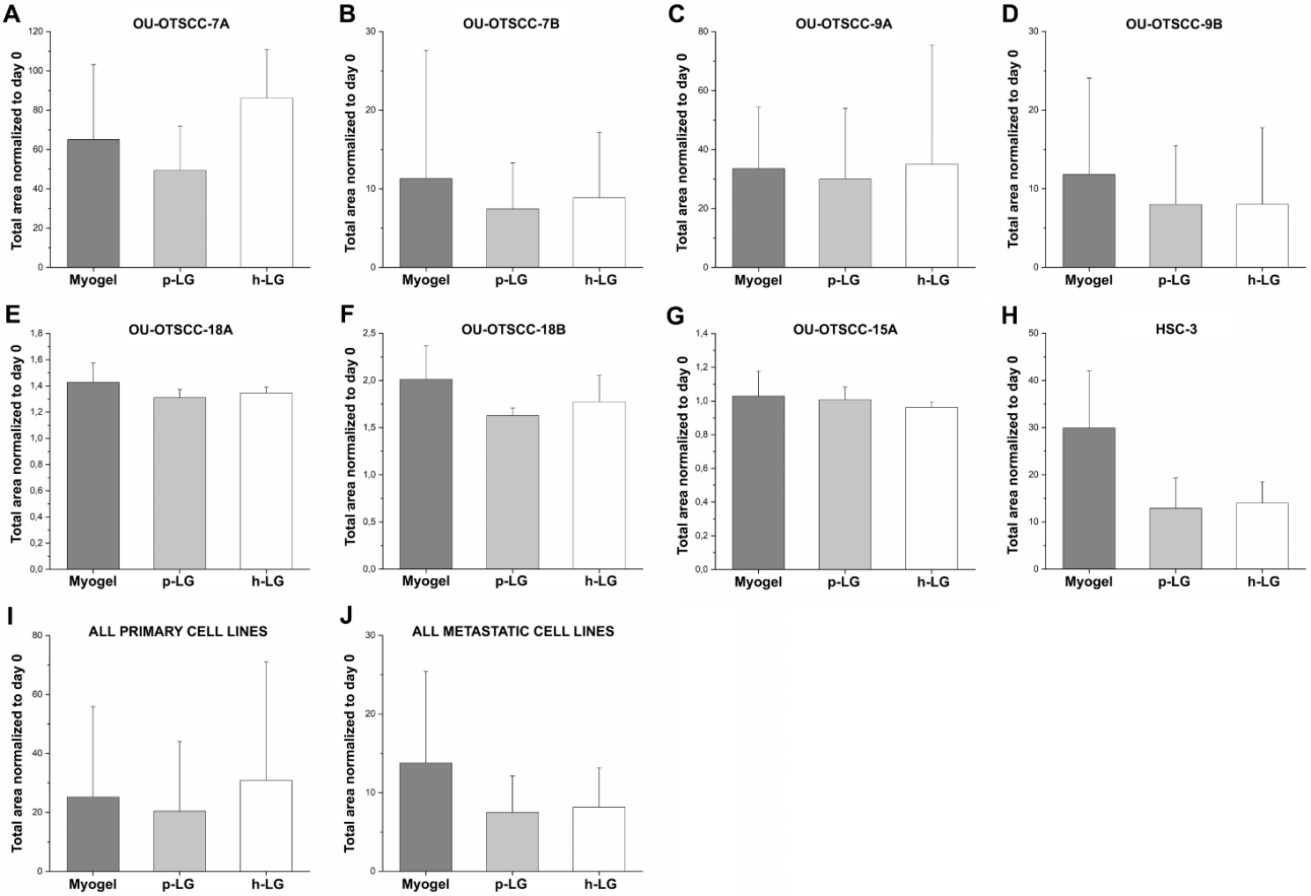
Spheroid invasion assay of OU-OTSCC (**A-G**), HSC-3 (**H**), combined primary (**I**) and combined metastatic (**J**) cell lines on different matrices. Spheroids cultured in ultra-low attachment 96-well round bottom plate wells were embedded in Myogel, porcine Lymphogel (p-LG) and human Lymphogel (h-LG) supplemented with fibrin. Results represent the total spheroid size at day 4 normalized with the day 0 size. The values represent the average ± SD of three to four independent experiments. For all “primary” and “metastatic” bars, the average results ± SD of four cell lines were combined. P-values were calculated with one-way analysis of variance (ANOVA) followed by Bonferroni correction

To study the effect of matrices on invasion of primary and metastatic cell lines, a spheroid invasion assay was conducted. The total spheroid size on day 4 was normalized with day 0, when spheroids were embedded in the matrix supplemented with fibrin. There was variation in spheroid size in different cell lines ranging from a 1 to 60 fold-change on day 4 compared with day 0 (Fig. 3). All matrices supported cell proliferation and invasion in all cell lines and without statistical differences. To test these matrices further, we performed a scratch wound migration and invasion assays on two additional primary and metastatic cell line pairs, UT-SCC-24A and -B and UT-SCC-42A and -B (Additional file 2: Fig. S3). Here also all matrices supported cell migration and invasion in these cell lines without any statistical differences.

### Protein composition of Lymphogels differs from that of myogel

**Fig. 4:**
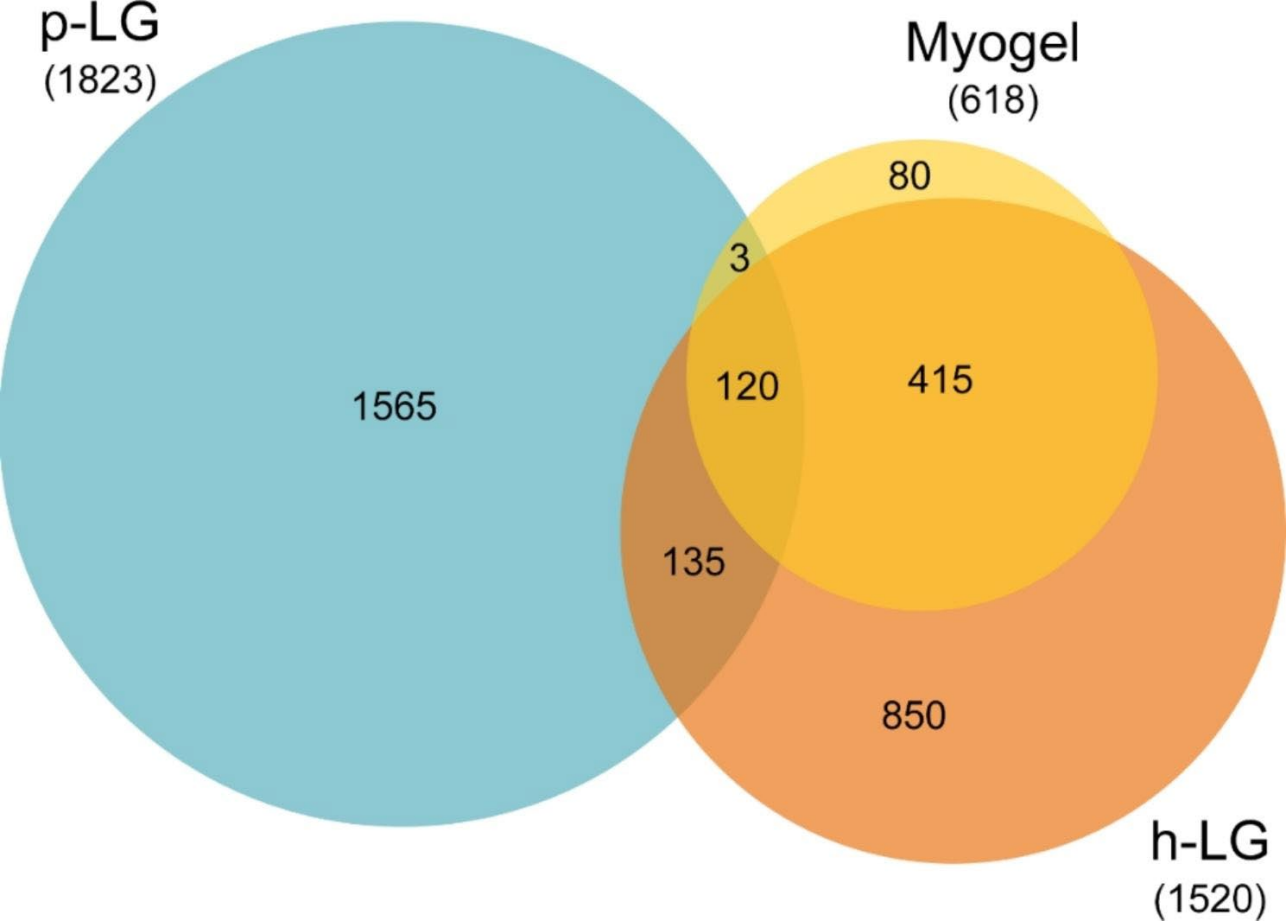
Comparison of identified proteins in different matrices. Protein compositions of Myogel, porcine Lymphogel (p-LG) and human Lymphogel (h-LG) were compared with each other

Proteomic analysis of Myogel revealed 618, p-LG 1823 and h-LG 1520 different proteins (Fig. 4). Comparison of Myogel with p-LG revealed 123, Myogel with h-LG 535 and porcine with human Lymphogels 255 common proteins. Comparison of all three matrices together revealed 120 common proteins.

**Fig. 5:**
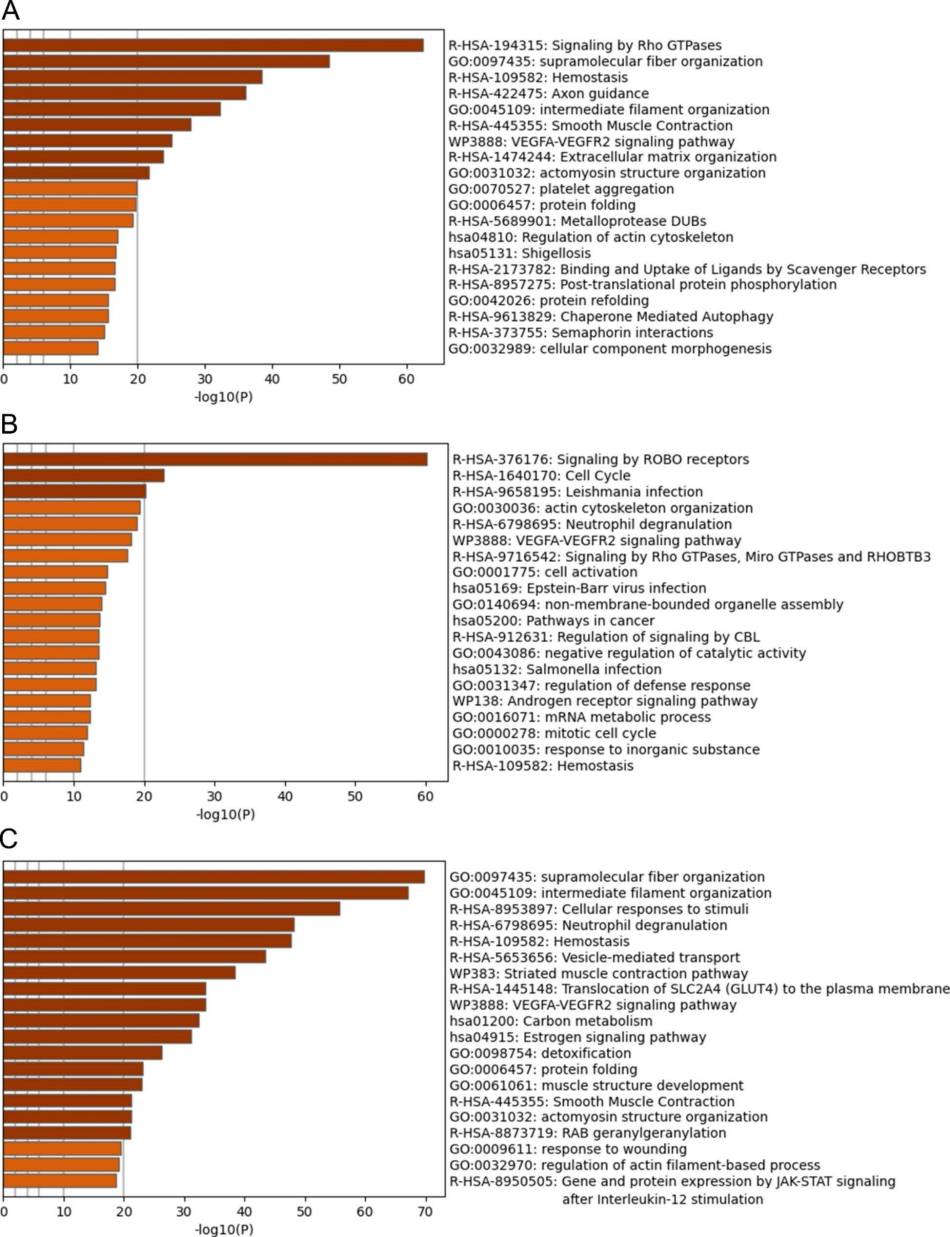
Metascape pathway and process enrichment analysis results of Myogel (**A**), p-LG (**B**) and h-LG (**C**). Bar graphs of enriched terms across input gene lists of each matrix (the top 20 clusters)

We used the Metascape Enriched Ontology Clustering on the proteomic data to explore the possible shared functional and mechanistic pathways between matrices. We obtained the top 20 clusters of processes and pathways in each matrix (Fig. 5). The analysis showed that Myogel and h-LG (Fig. 5A and C) both had enriched clusters of functional characteristics related to the cytoskeleton: supramolecular fibre organization, intermediate filament organization and actomyosin structure organization. In contrast, only Myogel contained proteins related to Rho GTPase-mediated signalling, extracellular matrix organization and metalloprotease DUBs (Fig. 5A). In h-LG, proteins related to cellular stimuli response, vesicle-mediated transport and JAK-STAT signalling were found (Fig. 5C). Neutrophil degranulation is active in both Lymphogels. In p-LG, one signalling pathway was particularly highlighted: signalling by ROBO receptors (Fig. 5B). VEGFA-VEGFR2 signalling pathway and haemostasis-related proteins were found in all matrices.

When the levels of shared protein entries between Myogel and h-LG were compared, structural proteins such as collagens were more abundant in Myogel. Adhesion and cytoskeleton-related pathways were highlighted in Metascape analysis of Myogel proteins (Additional file 1: Table S2, Additional file 2: Fig. S4). In comparison, in h-LG immune cell-related proteins such as immunoglobulins were more abundant, and pathways related to coagulation and immune system were highlighted.

### Human lymphogel contains unique adhesion proteins

**Fig. 6:**
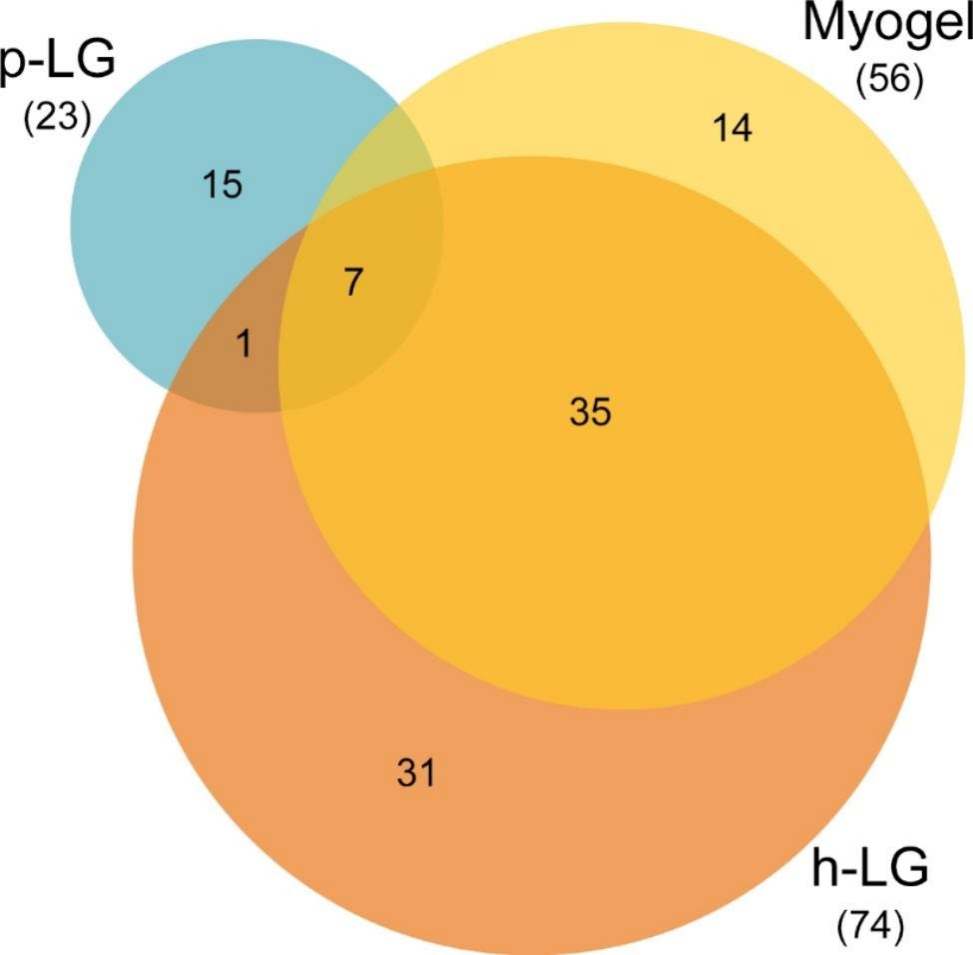
Summary of adhesion-related protein comparison in all three matrices: Myogel, porcine and human Lymphogels

We used the Gene Ontology database to identify the cell adhesion-mediating proteins that were present in each matrix. p-LG contained the smallest amount of adhesion proteins (23) and h-LG the largest (74) (Fig. 6). Myogel contained 56 adhesion-related proteins. Comparison of adhesion proteins revealed that Myogel and h-LG were the most similar, containing 42 common adhesion proteins (Fig. 6, Additional file 1: Table S4). All three matrices had seven proteins in common (Fig. 6, Additional file 1: Tables S3-S5), which were also the proteins shared by Myogel and p-LG (Fig. 6, Additional file 1: Table S3). In addition to these, both Lymphogels had one more protein in common (Fig. 6, Additional file 1: Table S5). Of the three matrices, human Lymphogel had the highest number of unique adhesion proteins (31) (Fig. 6, Additional file 1: Table S6), 12 of which (Additional file 1: Table S7) have been reported to be secreted or have subcellular location in cell junctions or extracellular matrix according to the UniProt database.

## Discussion

Results of cancer progression, metastatic processes and drug testing may often be misleading due to the lack of biologically relevant study matrices. Particularly, a LN-derived matrix that would be suitable for metastatic cell lines remains unavailable on the market. In this study, we established three tongue cancer cell line pairs: OU-OTSCC-7, -9, -18 including primary tumour (A) and their corresponding lymph node metastases (B) cells. In addition, we established one primary tumour cell line, OU-SCC-15, and used a commercial human metastatic OTSCC cell line, HSC-3, as a control for aggressive tumour behaviour. We used these cell lines to assess the effect of three different TME matrices, human uterine leiomyoma-derived Myogel and normal porcine neck LN- and human HNSCC patients’ pre-metastatic neck LN-derived Lymphogels, on carcinoma cells’ proliferation, adhesion, migration and invasion. We also investigated the protein composition of Myogel and both Lymphogels and compared them with each other to elucidate the differences in cell behaviour.

Primary and metastatic OTSCC cell lines showed similar cell viability on Myogel and both Lymphogels, confirming that all matrices had a protein composition that supports cell growth. In all primary-metastatic pairs, metastatic cells adhered more on human pre-metastatic LN-derived Lymphogel (h-LG). Although this difference between metastatic and primary cell adhesion on h-LG was clear, the same effect was not present on healthy porcine LN-derived Lymphogel (p-LG). When we studied OTSCC cells’ migration and invasion abilities on these different matrices by spheroid and wound healing assays, no clear matrix preference for primary or metastatic cell lines emerged. However, there was a non-significant trend that metastatic cells also invaded fastest on Myogel. This suggests that the protein composition, which induces the cell adhesion, may not support cell invasion. These results are in line with our previous findings that Myogel, compared with various other matrices, induces the invasion of cancer cells the most [17].

We performed the pathway and process enrichment analysis by Metascape to study the most active pathways in each matrix. Common enriched clusters of functional characteristics in Myogel and h-LG were cytoskeleton-related pathways and processes. Processes unique to Myogel seemingly associated with ECM and cell adhesion such as ECM reorganization, matrix metalloproteases (MMPs) and Rho GTPases. MMPs and Rho GTPases are linked to numerous key cell functions such as cell cycle progression, cell migration, malignant transformation, cell polarity, invasion and metastasis [28, 29]. In h-LG, various signalling and exosome-related pathways were highlighted in the Metascape analysis, which could be linked to pre-metastatic niche formation. Pre-metastatic niche formation has been introduced as a crucial step in the metastatic cascade and relies heavily on signalling, both molecular and exosomal, between tumour cells and the potential tissue site for metastasis [30]. As part of the innate immune system, neutrophils assure host defence via a range of pathways, e.g. neutrophil degranulation pathway [31]. The reason that this and other immune system and infection-related pathways were highlighted in human and porcine Lymphogels most likely is that they are derived from an immunologically active tissue, the lymph nodes. VEGFA-VEGFR2 signalling pathway and haemostasis-related proteins, which were found in all matrices, may represent vascularization of the source tissues. It also suggests that these matrices could support angiogenetic processes in vitro, since VEGFA-VEGFR2 pathway is associated with tumour progression by directly regulating angiogenesis, vascular permeability, cell migration and blood vessel-dependent metastasis [5, 32].

To focus on adhesion molecules for each matrix, a Gene Ontology functional annotation was applied to identify adhesion-related proteins in each matrix. p-LG contained the least adhesion proteins relative to Myogel and h-LG and had only a few proteins in common with these matrices. This might explain why a low number of cells attached to p-LG compared with other matrices. Myogel and h-LG shared 42 proteins, however, 31 adhesion proteins were only found in h-LG, in which we saw a difference between primary tumour and metastatic cell adhesion. Among unique adhesion proteins in h-LG were 12 proteins reported to localize outside the cell, in cell junctions or in extracellular matrix. These proteins included, for example, leucine-rich alpha-2-glycoprotein, annexin A1, annexin A2, galectin-3-binding protein, laminin subunit gamma-2 and plakophilin-2. These proteins have recently been linked to cancer progression. Overexpression of leucine-rich alpha-2-glycoprotein in malignant tumours has been shown to promote angiogenesis and EMT and inhibit apoptosis of cancer cells [33]. The expression of annexin A1 is associated with advanced stage of the disease, metastasis and differentiation status in head and neck cancers [34]. Annexin A2 might promote tumorigenesis by regulating multiple signal pathways inducing EMT [35]. Galectin-3-binding protein seems to participate in several pro-tumoural mechanisms in a variety of cancers [36]. Laminin subunit gamma-2 was upregulated in OTSCC and associated with an increased risk of recurrence and death [37]. Plakophilin-2 was shown to promote lung adenocarcinoma progression by enhancing EMT [38]. These extracellular adhesion proteins, which have been linked to cancer progression, might be important for adhesion of metastatic cancer cells to the TME in our in vitro adhesion assay, and probably also in vivo.

This study has potential limitations. Use of several more cell lines would have strengthened our conclusions on the effects of matrix on OTSCC cell adhesion and invasion patterns. However, due to unavailability of resources we were not able to include more than three primary-metastatic cancer cell lines to our experiments. Furthermore, since h-LG was done by combining material from more than one patient the variation in protein expression between patients was lost. In the future, a set of h-LGs produced from one patient could be analysed to find common proteins that might affect OTSCC cell behaviour. The wide range of standard deviations can be explained by high biological variation, which is natural to aggressive cancer cells, especially since the patient-derived cell lines we have used are not fully established. Furthermore, we report the combined averages of three or more experiments, which evidently increases the SD. Finally, the matrices could have been compared to widely used MatriGel, but since we have done this in our previous publications [9, 17, 18] and because we wanted to emphasize the human-derived ECMs, the comparison was excluded in this study.

## Conclusions

To increase the reliability of cancer cell experiments in vitro, it is critical to study the cells under biologically relevant conditions, understand the effects of TME and choose the appropriate matrix based on the cell lines and the type of assays. Here, we demonstrated that human HNSCC patients’ lymph node-derived matrix is suitable for studying metastatic oral tongue squamous cell carcinoma cells. It could be used for various 3D assays, e.g. spheroid invasion, in scratch wound migration and in invasion assays. In the future, it should be investigated whether this novel h-LG matrix could be useful in LN metastatic cancer’s drug screening and for personalized cancer medicine in vitro assays.

### Electronic supplementary material

Below is the link to the electronic supplementary material.

Additional file 1: Excel Tables

Additional file 2: Supplementary Figures

## Data Availability

Processed proteomics data can be found in the supplemental material of this article. The original datasets used during the study are available from the corresponding author upon reasonable request.

## Abbreviations

ECM: Extracellular matrix
EDTA: Ethylenediaminetetraacetic acid tetrasodium salt dihydrate
h-LG: Human lymph node-derived Lymphogel
HNSCC: Head and neck squamous cell carcinoma
LN: Lymph node
OTSCC: Oral tongue squamous cell carcinoma
PBS: Phosphate-buffered saline
p-LG: Porcine lymph node-derived Lymphogel
TME: Tumour microenvironment
Tris: Tris(hydroxymethyl)aminomethane
