# Supplementary material for: Novel human lymph node-derived matrix supports the adhesion of metastatic oral carcinoma cells
Source: BMC Cancer. 2023 Aug 14;23:750. doi: 10.1186/s12885-023-11275-6 (PMC10424355; doi:10.1186/s12885-023-11275-6)
Supplement: Supplementary file 2 — Additional file 2: Supplementary Figures [file 12885_2023_11275_MOESM2_ESM.docx]

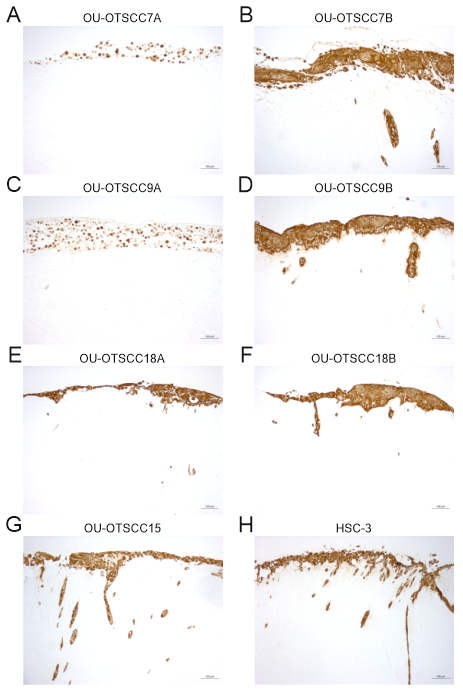


Fig. S1. Myoma organotypic cultures of OU-OTSCC and HSC-3 cell lines. Cells were cultured on top of myoma discs for 18-20 days. Paraffin-embedded sections were immunostained with the epithelial marker pancytokeratin (AE1/AE3, brown). Original magnification, 100x.


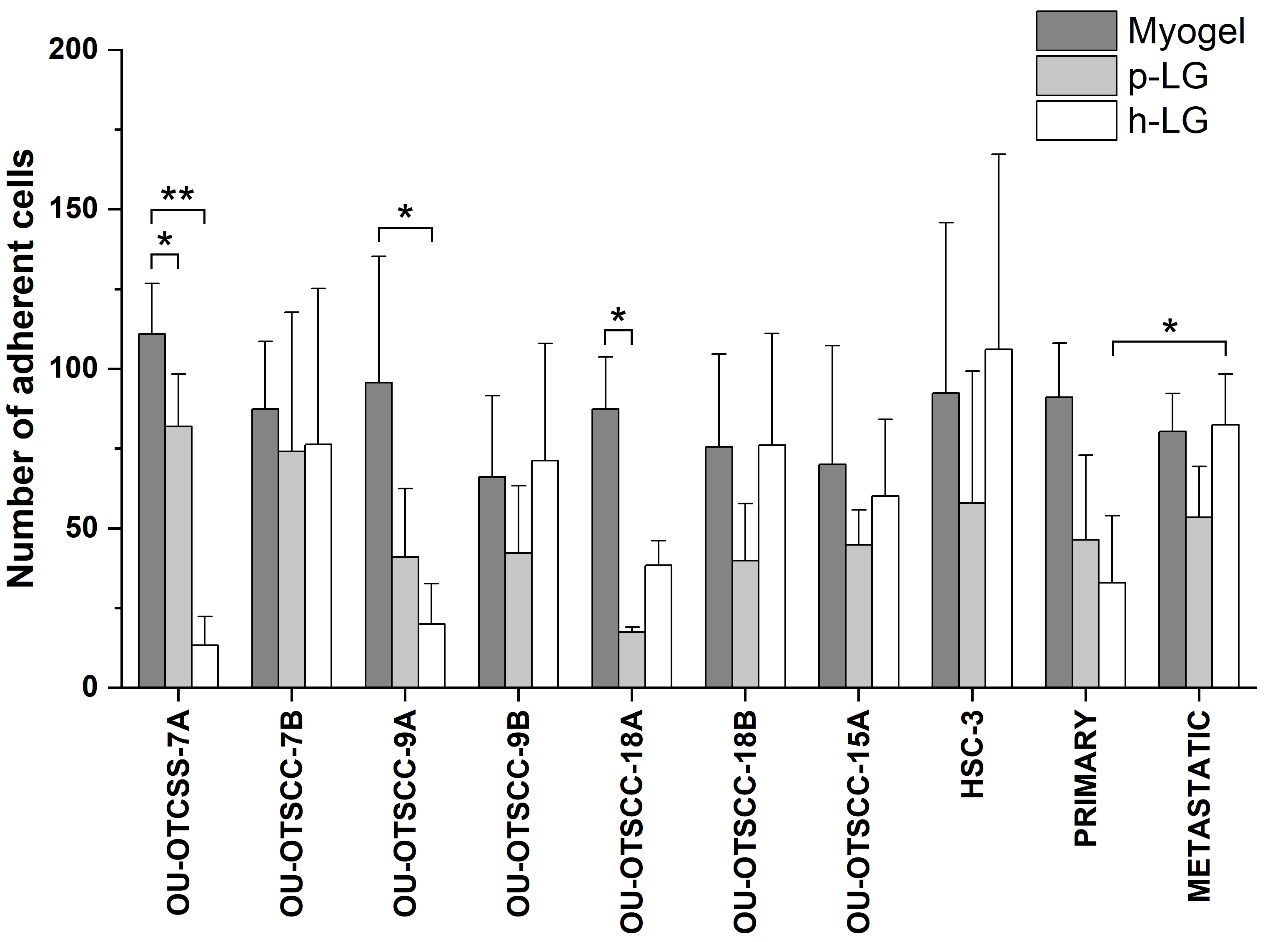


Fig. S2. Adhesion of OU-OTSCC and HSC-3 cell lines on different matrices. Cells were allowed to attach for 2 h in wells coated with Myogel, porcine Lymphogel (p-LG) and human Lymphogel (h-LG) after which cells were fixed, stained with crystal violet and counted. The values of the cell lines represent the average ± SD of three independent experiments. For “primary” and “metastatic” bars, the average results ± SD of four cell lines were combined. P-values were calculated with one-way analysis of variance (ANOVA) followed by Bonferroni correction and independent samples T-test. *P<0.05, **P<0.001.


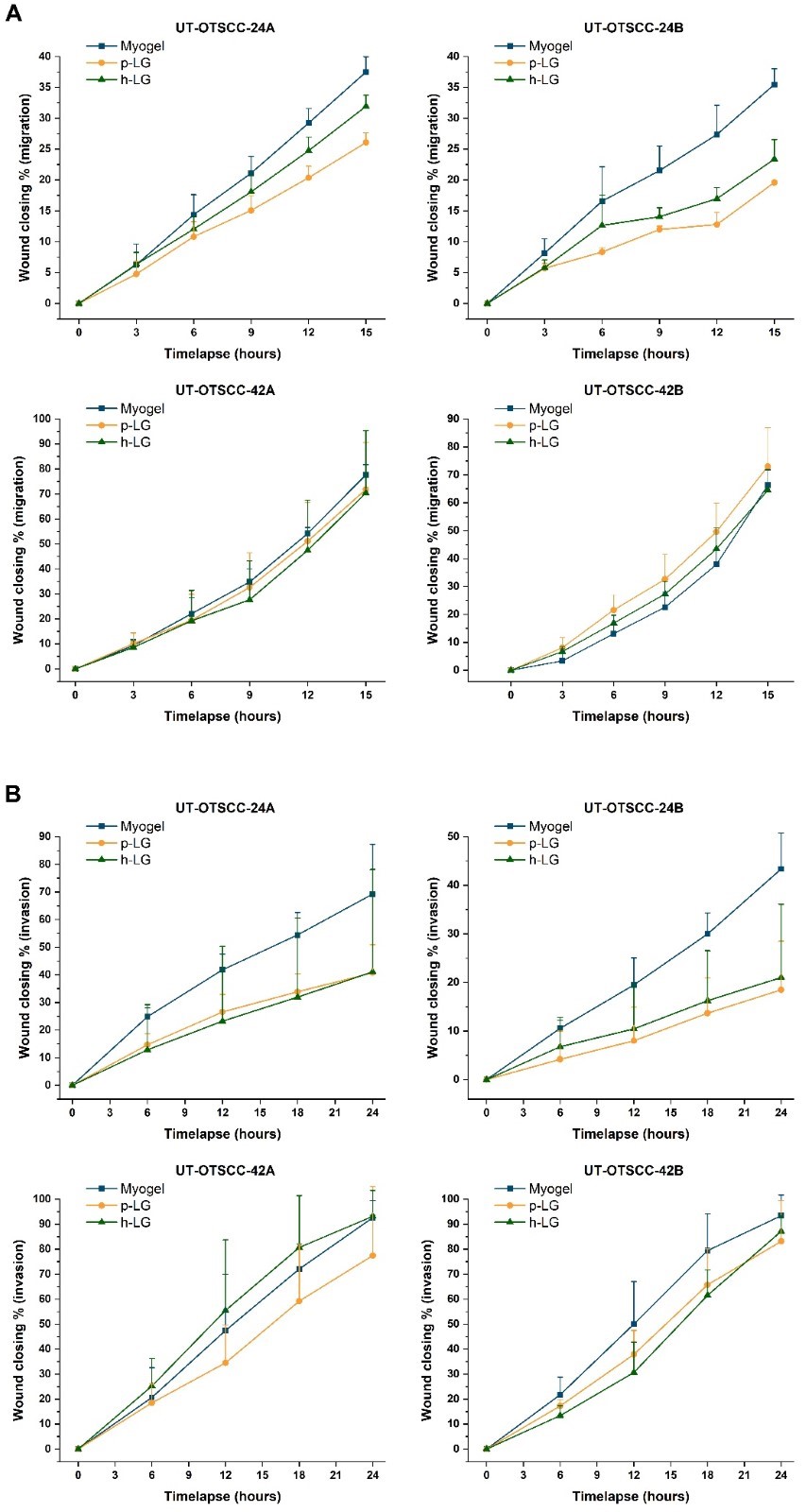


Fig. S3. Cell migration and invasion scratch wound assays performed using primary and metastatic UT-OTSCC cell lines. Quantification of cell migration on Myogel, porcine Lymphogel (p-LG) and human Lymphogel (h-LG) (A). Cell invasion through Myogel-collagen, porcine Lymphogel-collagen and human Lymphogel-collagen matrices (B). Data are presented as invasion curves as means ± SD of three independent experiments, each at least in triplicate.


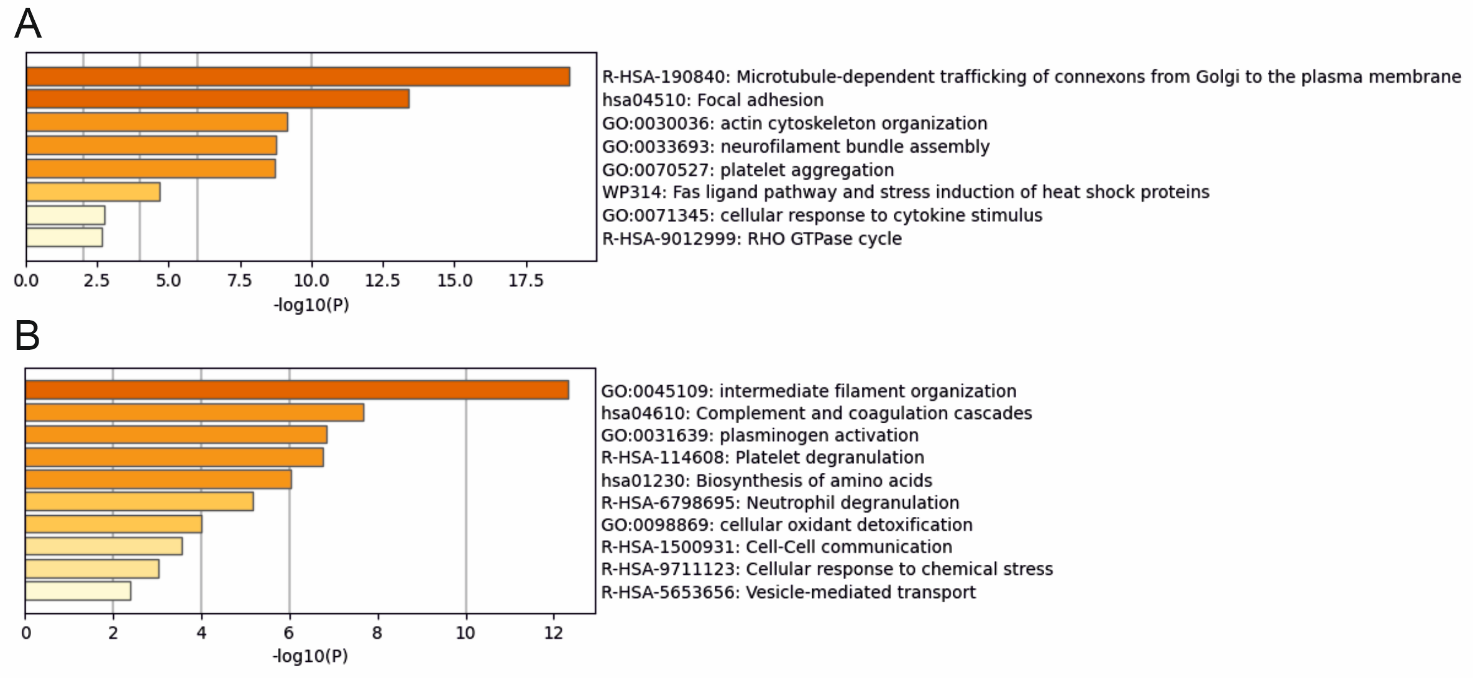


Fig. S4 Metascape pathway and process enrichment analysis of shared proteins between human Myogel and Lymphogel that have higher expression in human Myogel (A) or in human Lymphogel (B).
